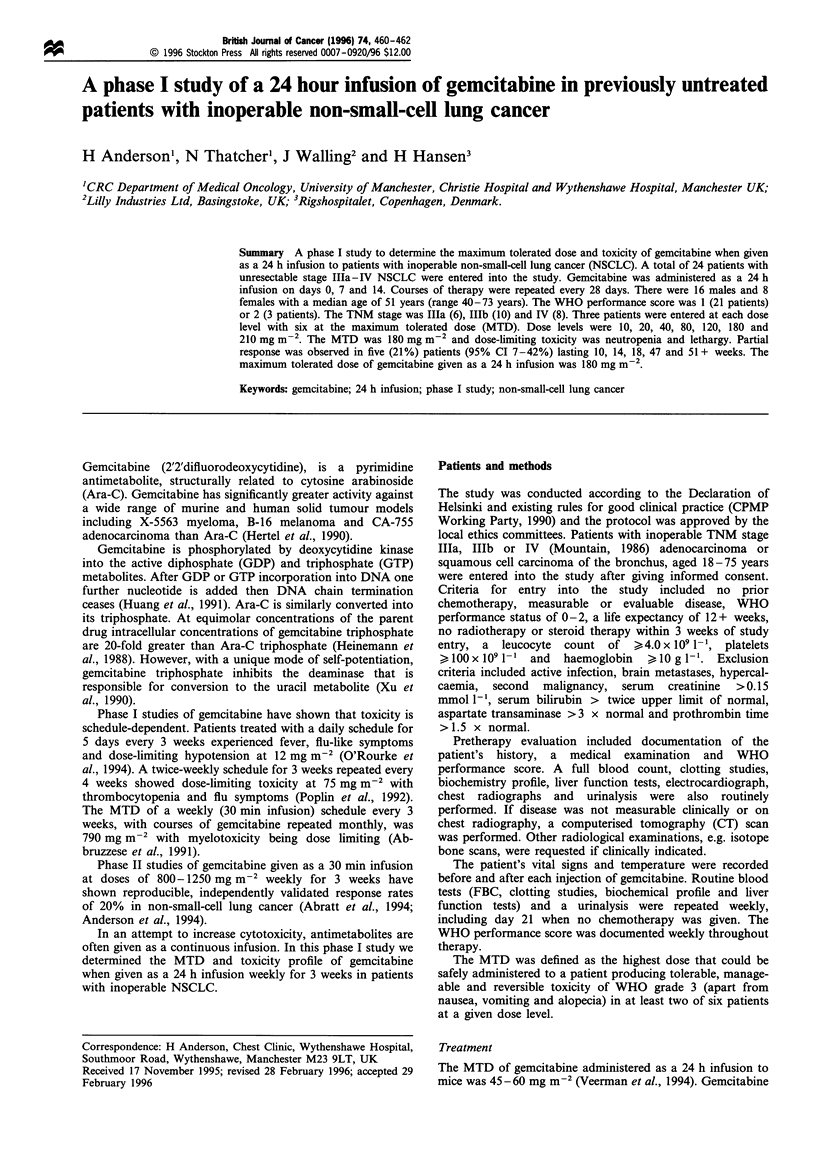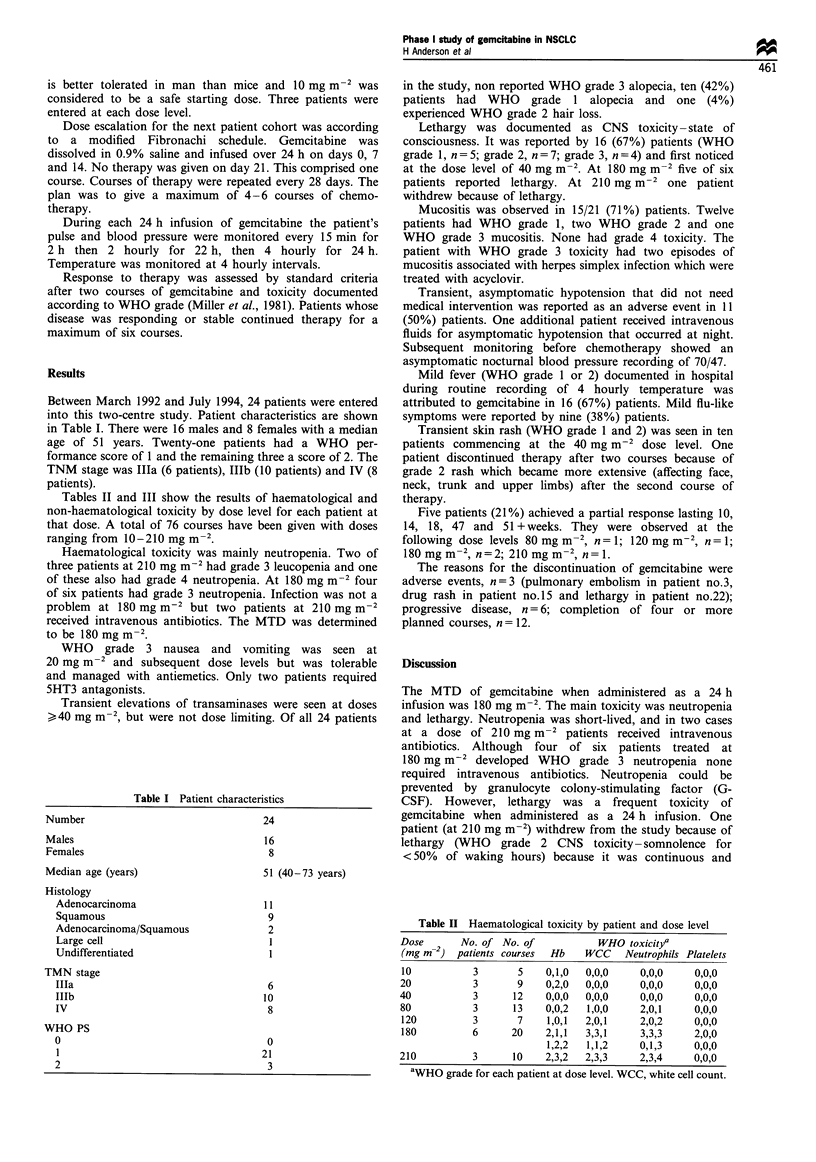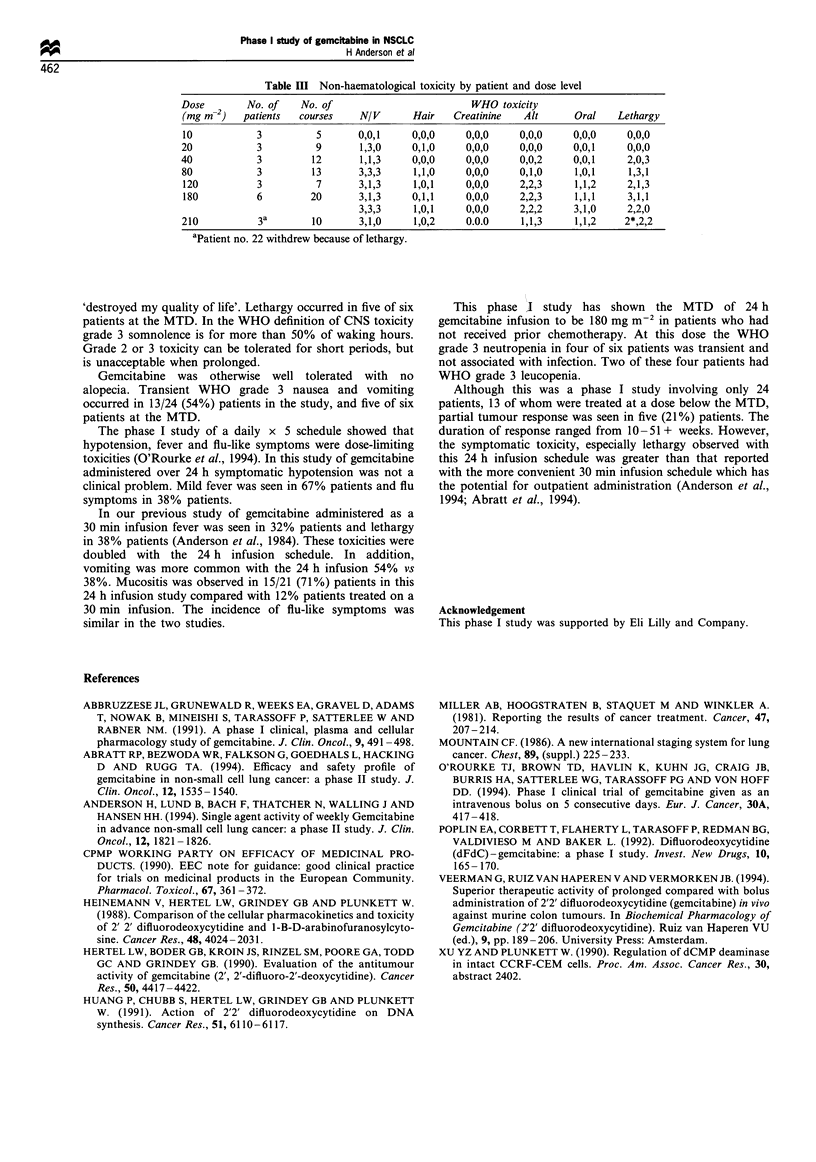# A phase I study of a 24 hour infusion of gemcitabine in previously untreated patients with inoperable non-small-cell lung cancer.

**DOI:** 10.1038/bjc.1996.382

**Published:** 1996-08

**Authors:** H. Anderson, N. Thatcher, J. Walling, H. Hansen

**Affiliations:** CRC Department of Medical Oncology, University of Manchester, Christie Hospital and Wythenshawe Hospital, UK.

## Abstract

A phase I study to determine the maximum tolerated dose and toxicity of gemcitabine when given as a 24 h infusion to patients with inoperable non-small-cell lung cancer (NSCLC). A total of 24 patients with unresectable stage IIIa-IV NSCLC were entered into the study. Gemcitabine was administered as a 24 h infusion on days 0, 7 and 14. Courses of therapy were repeated every 28 days. There were 16 males and 8 females with a median age of 51 years (range 40-73 years). The WHO performance score was 1 (21 patients) or 2 (3 patients). The TNM stage was IIIa (6), IIIb (10) and IV (8). Three patients were entered at each dose level with six at the maximum tolerated dose (MTD). Dose levels were 10, 20, 40, 80, 120, 180 and 210 mg m-2. The MTD was 180 mg m-2 and dose-limiting toxicity was neutropenia and lethargy. Partial response was observed in five (21%) patients (95% CI 7-42%) lasting 10, 14, 18, 47 and 51 + weeks. The maximum tolerated dose of gemcitabine given as a 24 h infusion was 180 mg m-2.


British Journal of Cancer (1996) 74, 460–462
© 1996 Stockton Press All rights reserved 0007–0920/96 $12.00

### A phase I study of a 24 hour infusion of gemcitabine in previously untreated patients with inoperable non-small-cell lung cancer

H Anderson[1], N Thatcher[1], J Walling[2] and H Hansen[3]

[1]*CRC Department of Medical Oncology, University of Manchester, Christie Hospital and Wythenshawe Hospital, Manchester UK;* [2]*Lilly Industries Ltd, Basingstoke, UK;* [3]*Rigshospitalet, Copenhagen, Denmark.*

**Summary** A phase I study to determine the maximum tolerated dose and toxicity of gemcitabine when given as a 24 h infusion to patients with inoperable non-small-cell lung cancer (NSCLC). A total of 24 patients with unresectable stage IIIa–IV NSCLC were entered into the study. Gemcitabine was administered as a 24 h infusion on days 0, 7 and 14. Courses of therapy were repeated every 28 days. There were 16 males and 8 females with a median age of 51 years (range 40–73 years). The WHO performance score was 1 (21 patients) or 2 (3 patients). The TNM stage was IIIa (6), IIIb (10) and IV (8). Three patients were entered at each dose level with six at the maximum tolerated dose (MTD). Dose levels were 10, 20, 40, 80, 120, 180 and 210 mg $m^{-2}$. The MTD was 180 mg $m^{-2}$ and dose-limiting toxicity was neutropenia and lethargy. Partial response was observed in five (21%) patients (95% CI 7–42%) lasting 10, 14, 18, 47 and 51+ weeks. The maximum tolerated dose of gemcitabine given as a 24 h infusion was 180 mg $m^{-2}$.

**Keywords:** gemcitabine; 24 h infusion; phase I study; non-small-cell lung cancer

Gemcitabine (2′2′difluorodeoxycytidine), is a pyrimidine antimetabolite, structurally related to cytosine arabinoside (Ara-C). Gemcitabine has significantly greater activity against a wide range of murine and human solid tumour models including X-5563 myeloma, B-16 melanoma and CA-755 adenocarcinoma than Ara-C (Hertel *et al.*, 1990).

Gemcitabine is phosphorylated by deoxycytidine kinase into the active diphosphate (GDP) and triphosphate (GTP) metabolites. After GDP or GTP incorporation into DNA one further nucleotide is added then DNA chain termination ceases (Huang *et al.*, 1991). Ara-C is similarly converted into its triphosphate. At equimolar concentrations of the parent drug intracellular concentrations of gemcitabine triphosphate are 20-fold greater than Ara-C triphosphate (Heinemann *et al.*, 1988). However, with a unique mode of self-potentiation, gemcitabine triphosphate inhibits the deaminase that is responsible for conversion to the uracil metabolite (Xu *et al.*, 1990).

Phase I studies of gemcitabine have shown that toxicity is schedule-dependent. Patients treated with a daily schedule for 5 days every 3 weeks experienced fever, flu-like symptoms and dose-limiting hypotension at 12 mg $m^{-2}$ (O'Rourke *et al.*, 1994). A twice-weekly schedule for 3 weeks repeated every 4 weeks showed dose-limiting toxicity at 75 mg $m^{-2}$ with thrombocytopenia and flu symptoms (Poplin *et al.*, 1992). The MTD of a weekly (30 min infusion) schedule every 3 weeks, with courses of gemcitabine repeated monthly, was 790 mg $m^{-2}$ with myelotoxicity being dose limiting (Abbruzzese *et al.*, 1991).

Phase II studies of gemcitabine given as a 30 min infusion at doses of 800–1250 mg $m^{-2}$ weekly for 3 weeks have shown reproducible, independently validated response rates of 20% in non-small-cell lung cancer (Abratt *et al.*, 1994; Anderson *et al.*, 1994).

In an attempt to increase cytotoxicity, antimetabolites are often given as a continuous infusion. In this phase I study we determined the MTD and toxicity profile of gemcitabine when given as a 24 h infusion weekly for 3 weeks in patients with inoperable NSCLC.

Correspondence: H Anderson, Chest Clinic, Wythenshawe Hospital, Southmoor Road, Wythenshawe, Manchester M23 9LT, UK
Received 17 November 1995; revised 28 February 1996; accepted 29 February 1996

### Patients and methods

The study was conducted according to the Declaration of Helsinki and existing rules for good clinical practice (CPMP Working Party, 1990) and the protocol was approved by the local ethics committees. Patients with inoperable TNM stage IIIa, IIIb or IV (Mountain, 1986) adenocarcinoma or squamous cell carcinoma of the bronchus, aged 18–75 years were entered into the study after giving informed consent. Criteria for entry into the study included no prior chemotherapy, measurable or evaluable disease, WHO performance status of 0–2, a life expectancy of 12+ weeks, no radiotherapy or steroid therapy within 3 weeks of study entry, a leucocyte count of $\geqslant 4.0 \times 10^9\ l^{-1}$, platelets $\geqslant 100 \times 10^9\ l^{-1}$ and haemoglobin $\geqslant 10\ g\ l^{-1}$. Exclusion criteria included active infection, brain metastases, hypercalcaemia, second malignancy, serum creatinine >0.15 mmol $l^{-1}$, serum bilirubin > twice upper limit of normal, aspartate transaminase >3 × normal and prothrombin time >1.5 × normal.

Pretherapy evaluation included documentation of the patient's history, a medical examination and WHO performance score. A full blood count, clotting studies, biochemistry profile, liver function tests, electrocardiograph, chest radiographs and urinalysis were also routinely performed. If disease was not measurable clinically or on chest radiography, a computerised tomography (CT) scan was performed. Other radiological examinations, e.g. isotope bone scans, were requested if clinically indicated.

The patient's vital signs and temperature were recorded before and after each injection of gemcitabine. Routine blood tests (FBC, clotting studies, biochemical profile and liver function tests) and a urinalysis were repeated weekly, including day 21 when no chemotherapy was given. The WHO performance score was documented weekly throughout therapy.

The MTD was defined as the highest dose that could be safely administered to a patient producing tolerable, manageable and reversible toxicity of WHO grade 3 (apart from nausea, vomiting and alopecia) in at least two of six patients at a given dose level.

### *Treatment*

The MTD of gemcitabine administered as a 24 h infusion to mice was 45–60 mg $m^{-2}$ (Veerman *et al.*, 1994). Gemcitabine is better tolerated in man than mice and 10 mg $m^{-2}$ was considered to be a safe starting dose. Three patients were entered at each dose level.

Dose escalation for the next patient cohort was according to a modified Fibronachi schedule. Gemcitabine was dissolved in 0.9% saline and infused over 24 h on days 0, 7 and 14. No therapy was given on day 21. This comprised one course. Courses of therapy were repeated every 28 days. The plan was to give a maximum of 4–6 courses of chemotherapy.

During each 24 h infusion of gemcitabine the patient's pulse and blood pressure were monitored every 15 min for 2 h then 2 hourly for 22 h, then 4 hourly for 24 h. Temperature was monitored at 4 hourly intervals.

Response to therapy was assessed by standard criteria after two courses of gemcitabine and toxicity documented according to WHO grade (Miller *et al.*, 1981). Patients whose disease was responding or stable continued therapy for a maximum of six courses.

### Results

Between March 1992 and July 1994, 24 patients were entered into this two-centre study. Patient characteristics are shown in Table I. There were 16 males and 8 females with a median age of 51 years. Twenty-one patients had a WHO performance score of 1 and the remaining three a score of 2. The TNM stage was IIIa (6 patients), IIIb (10 patients) and IV (8 patients).

Tables II and III show the results of haematological and non-haematological toxicity by dose level for each patient at that dose. A total of 76 courses have been given with doses ranging from 10–210 mg $m^{-2}$.

Haematological toxicity was mainly neutropenia. Two of three patients at 210 mg $m^{-2}$ had grade 3 leucopenia and one of these also had grade 4 neutropenia. At 180 mg $m^{-2}$ four of six patients had grade 3 neutropenia. Infection was not a problem at 180 mg $m^{-2}$ but two patients at 210 mg $m^{-2}$ received intravenous antibiotics. The MTD was determined to be 180 mg $m^{-2}$.

WHO grade 3 nausea and vomiting was seen at 20 mg $m^{-2}$ and subsequent dose levels but was tolerable and managed with antiemetics. Only two patients required 5HT3 antagonists.

Transient elevations of transaminases were seen at doses ⩾40 mg $m^{-2}$, but were not dose limiting. Of all 24 patients in the study, non reported WHO grade 3 alopecia, ten (42%) patients had WHO grade 1 alopecia and one (4%) experienced WHO grade 2 hair loss.

Lethargy was documented as CNS toxicity–state of consciousness. It was reported by 16 (67%) patients (WHO grade 1, $n=5$; grade 2, $n=7$; grade 3, $n=4$) and first noticed at the dose level of 40 mg $m^{-2}$. At 180 mg $m^{-2}$ five of six patients reported lethargy. At 210 mg $m^{-2}$ one patient withdrew because of lethargy.

Mucositis was observed in 15/21 (71%) patients. Twelve patients had WHO grade 1, two WHO grade 2 and one WHO grade 3 mucositis. None had grade 4 toxicity. The patient with WHO grade 3 toxicity had two episodes of mucositis associated with herpes simplex infection which were treated with acyclovir.

Transient, asymptomatic hypotension that did not need medical intervention was reported as an adverse event in 11 (50%) patients. One additional patient received intravenous fluids for asymptomatic hypotension that occurred at night. Subsequent monitoring before chemotherapy showed an asymptomatic nocturnal blood pressure recording of 70/47.

Mild fever (WHO grade 1 or 2) documented in hospital during routine recording of 4 hourly temperature was attributed to gemcitabine in 16 (67%) patients. Mild flu-like symptoms were reported by nine (38%) patients.

Transient skin rash (WHO grade 1 and 2) was seen in ten patients commencing at the 40 mg $m^{-2}$ dose level. One patient discontinued therapy after two courses because of grade 2 rash which became more extensive (affecting face, neck, trunk and upper limbs) after the second course of therapy.

Five patients (21%) achieved a partial response lasting 10, 14, 18, 47 and 51+weeks. They were observed at the following dose levels 80 mg $m^{-2}$, $n=1$; 120 mg $m^{-2}$, $n=1$; 180 mg $m^{-2}$, $n=2$; 210 mg $m^{-2}$, $n=1$.

The reasons for the discontinuation of gemcitabine were adverse events, $n=3$ (pulmonary embolism in patient no.3, drug rash in patient no.15 and lethargy in patient no.22); progressive disease, $n=6$; completion of four or more planned courses, $n=12$.

### Discussion

The MTD of gemcitabine when administered as a 24 h infusion was 180 mg $m^{-2}$. The main toxicity was neutropenia and lethargy. Neutropenia was short-lived, and in two cases at a dose of 210 mg $m^{-2}$ patients received intravenous antibiotics. Although four of six patients treated at 180 mg $m^{-2}$ developed WHO grade 3 neutropenia none required intravenous antibiotics. Neutropenia could be prevented by granulocyte colony-stimulating factor (G-CSF). However, lethargy was a frequent toxicity of gemcitabine when administered as a 24 h infusion. One patient (at 210 mg $m^{-2}$) withdrew from the study because of lethargy (WHO grade 2 CNS toxicity–somnolence for <50% of waking hours) because it was continuous and

**Table I** Patient characteristics

| | |
|---|---|
| Number | 24 |
| Males | 16 |
| Females | 8 |
| Median age (years) | 51 (40–73 years) |
| Histology | |
| Adenocarcinoma | 11 |
| Squamous | 9 |
| Adenocarcinoma/Squamous | 2 |
| Large cell | 1 |
| Undifferentiated | 1 |
| TMN stage | |
| IIIa | 6 |
| IIIb | 10 |
| IV | 8 |
| WHO PS | |
| 0 | 0 |
| 1 | 21 |
| 2 | 3 |

**Table II** Haematological toxicity by patient and dose level

| Dose (mg $m^{-2}$) | No. of patients | No. of courses | Hb | WHO toxicity[a] WCC | Neutrophils | Platelets |
|---|---|---|---|---|---|---|
| 10 | 3 | 5 | 0,1,0 | 0,0,0 | 0,0,0 | 0,0,0 |
| 20 | 3 | 9 | 0,2,0 | 0,0,0 | 0,0,0 | 0,0,0 |
| 40 | 3 | 12 | 0,0,0 | 0,0,0 | 0,0,0 | 0,0,0 |
| 80 | 3 | 13 | 0,0,2 | 1,0,0 | 2,0,1 | 0,0,0 |
| 120 | 3 | 7 | 1,0,1 | 2,0,1 | 2,0,2 | 0,0,0 |
| 180 | 6 | 20 | 2,1,1 | 3,3,1 | 3,3,3 | 2,0,0 |
| | | | 1,2,2 | 1,1,2 | 0,1,3 | 0,0,0 |
| 210 | 3 | 10 | 2,3,2 | 2,3,3 | 2,3,4 | 0,0,0 |

[a]WHO grade for each patient at dose level. WCC, white cell count.

**Table III** Non-haematological toxicity by patient and dose level

| *Dose (mg m$^{-2}$)* | *No. of patients* | *No. of courses* | *WHO toxicity* | | | | | |
|---|---|---|---|---|---|---|---|---|
| | | | *N/V* | *Hair* | *Creatinine* | *Alt* | *Oral* | *Lethargy* |
| 10 | 3 | 5 | 0,0,1 | 0,0,0 | 0,0,0 | 0,0,0 | 0,0,0 | 0,0,0 |
| 20 | 3 | 9 | 1,3,0 | 0,1,0 | 0,0,0 | 0,0,0 | 0,0,1 | 0,0,0 |
| 40 | 3 | 12 | 1,1,3 | 0,0,0 | 0,0,0 | 0,0,2 | 0,0,1 | 2,0,3 |
| 80 | 3 | 13 | 3,3,3 | 1,1,0 | 0,0,0 | 0,1,0 | 1,0,1 | 1,3,1 |
| 120 | 3 | 7 | 3,1,3 | 1,0,1 | 0,0,0 | 2,2,3 | 1,1,2 | 2,1,3 |
| 180 | 6 | 20 | 3,1,3 | 0,1,1 | 0,0,0 | 2,2,3 | 1,1,1 | 3,1,1 |
| | | | 3,3,3 | 1,0,1 | 0,0,0 | 2,2,2 | 3,1,0 | 2,2,0 |
| 210 | 3[a] | 10 | 3,1,0 | 1,0,2 | 0.0.0 | 1,1,3 | 1,1,2 | 2*,2,2 |

[a]Patient no. 22 withdrew because of lethargy.

'destroyed my quality of life'. Lethargy occurred in five of six patients at the MTD. In the WHO definition of CNS toxicity grade 3 somnolence is for more than 50% of waking hours. Grade 2 or 3 toxicity can be tolerated for short periods, but is unacceptable when prolonged.

Gemcitabine was otherwise well tolerated with no alopecia. Transient WHO grade 3 nausea and vomiting occurred in 13/24 (54%) patients in the study, and five of six patients at the MTD.

The phase I study of a daily × 5 schedule showed that hypotension, fever and flu-like symptoms were dose-limiting toxicities (O'Rourke *et al.*, 1994). In this study of gemcitabine administered over 24 h symptomatic hypotension was not a clinical problem. Mild fever was seen in 67% patients and flu symptoms in 38% patients.

In our previous study of gemcitabine administered as a 30 min infusion fever was seen in 32% patients and lethargy in 38% patients (Anderson *et al.*, 1984). These toxicities were doubled with the 24 h infusion schedule. In addition, vomiting was more common with the 24 h infusion 54% *vs* 38%. Mucositis was observed in 15/21 (71%) patients in this 24 h infusion study compared with 12% patients treated on a 30 min infusion. The incidence of flu-like symptoms was similar in the two studies.

This phase I study has shown the MTD of 24 h gemcitabine infusion to be 180 mg m$^{-2}$ in patients who had not received prior chemotherapy. At this dose the WHO grade 3 neutropenia in four of six patients was transient and not associated with infection. Two of these four patients had WHO grade 3 leucopenia.

Although this was a phase I study involving only 24 patients, 13 of whom were treated at a dose below the MTD, partial tumour response was seen in five (21%) patients. The duration of response ranged from 10–51+ weeks. However, the symptomatic toxicity, especially lethargy observed with this 24 h infusion schedule was greater than that reported with the more convenient 30 min infusion schedule which has the potential for outpatient administration (Anderson *et al.*, 1994; Abratt *et al.*, 1994).

**Acknowledgement**
This phase I study was supported by Eli Lilly and Company.

### References

ABBRUZZESE JL, GRUNEWALD R, WEEKS EA, GRAVEL D, ADAMS T, NOWAK B, MINEISHI S, TARASSOFF P, SATTERLEE W AND RABNER NM. (1991). A phase I clinical, plasma and cellular pharmacology study of gemcitabine. *J. Clin. Oncol.*, **9**, 491–498.

ABRATT RP, BEZWODA WR, FALKSON G, GOEDHALS L, HACKING D AND RUGG TA. (1994). Efficacy and safety profile of gemcitabine in non-small cell lung cancer: a phase II study. *J. Clin. Oncol.*, **12**, 1535–1540.

ANDERSON H, LUND B, BACH F, THATCHER N, WALLING J AND HANSEN HH. (1994). Single agent activity of weekly Gemcitabine in advance non-small cell lung cancer: a phase II study. *J. Clin. Oncol.*, **12**, 1821–1826.

CPMP WORKING PARTY ON EFFICACY OF MEDICINAL PRODUCTS. (1990). EEC note for guidance: good clinical practice for trials on medicinal products in the European Community. *Pharmacol. Toxicol.*, **67**, 361–372.

HEINEMANN V, HERTEL LW, GRINDEY GB AND PLUNKETT W. (1988). Comparison of the cellular pharmacokinetics and toxicity of 2′ 2′ difluorodeoxycytidine and 1-B-D-arabinofuranosylcytosine. *Cancer Res.*, **48**, 4024–2031.

HERTEL LW, BODER GB, KROIN JS, RINZEL SM, POORE GA, TODD GC AND GRINDEY GB. (1990). Evaluation of the antitumour activity of gemcitabine (2′, 2′-difluoro-2′-deoxycytidine). *Cancer Res.*, **50**, 4417–4422.

HUANG P, CHUBB S, HERTEL LW, GRINDEY GB AND PLUNKETT W. (1991). Action of 2′2′ difluorodeoxycytidine on DNA synthesis. *Cancer Res.*, **51**, 6110–6117.

MILLER AB, HOOGSTRATEN B, STAQUET M AND WINKLER A. (1981). Reporting the results of cancer treatment. *Cancer*, **47**, 207–214.

MOUNTAIN CF. (1986). A new international staging system for lung cancer. *Chest*, **89**, (suppl.) 225–233.

O'ROURKE TJ, BROWN TD, HAVLIN K, KUHN JG, CRAIG JB, BURRIS HA, SATTERLEE WG, TARASSOFF PG AND VON HOFF DD. (1994). Phase I clinical trial of gemcitabine given as an intravenous bolus on 5 consecutive days. *Eur. J. Cancer*, **30A**, 417–418.

POPLIN EA, CORBETT T, FLAHERTY L, TARASOFF P, REDMAN BG, VALDIVIESO M AND BAKER L. (1992). Difluorodeoxycytidine (dFdC)-gemcitabine: a phase I study. *Invest. New Drugs*, **10**, 165–170.

VEERMAN G, RUIZ VAN HAPEREN V AND VERMORKEN JB. (1994). Superior therapeutic activity of prolonged compared with bolus administration of 2′2′ difluorodeoxycytidine (gemcitabine) *in vivo* against murine colon tumours. In *Biochemical Pharmacology of Gemcitabine (2′2′* difluorodeoxycytidine). Ruiz van Haperen VU (ed.), **9**, pp. 189–206. University Press: Amsterdam.

XU YZ AND PLUNKETT W. (1990). Regulation of dCMP deaminase in intact CCRF-CEM cells. *Proc. Am. Assoc. Cancer Res.*, **30**, abstract 2402.